# Biomimetic Intrafibrillar Mineralization of Type I Collagen with Intermediate Precursors-loaded Mesoporous Carriers

**DOI:** 10.1038/srep11199

**Published:** 2015-06-08

**Authors:** Wei Zhang, Xiao-juan Luo, Li-na Niu, Hong-ye Yang, Cynthia K.Y. Yiu, Tian-da Wang, Li-qun Zhou, Jing Mao, Cui Huang, David H. Pashley, Franklin R. Tay

**Affiliations:** 1 Tongji Hospital, Huazhong University of Science and Technology, Wuhan, PR China; 2 State Key Laboratory of Military Stomatology, Department of Prosthodontics, School of Stomatology, The Fourth Military Medical University, Xi’an, PR China; 3 Wuhan University, Wuhan, PR China; 4 University of Hong Kong, Hong Kong SAR, PR China; 5 Peking University School and Hospital of Stomatology, Beijing, PR China; 6 Georgia Regents University, Augusta, Georgia, 30912-1129, USA

## Abstract

Limited continuous replenishment of the mineralization medium is a restriction for *in-situ* solution-based remineralization of hypomineralized body tissues. Here, we report a process that generated amine-functionalized mesoporous silica nanoparticles for sustained release of biomimetic analog-stabilized amorphous calcium phosphate precursors. Both two-dimensional and three-dimensional collagen models can be intrafibrillarly mineralized with these released fluidic intermediate precursors. This represents an important advance in the translation of biomineralization concepts into regimes for *in-situ* remineralization of bone and teeth.

The non-classical pathway of particle-mediated crystallization has gained momentum in being accepted as the mechanism for intrafibrillar mineralization of collagen with calcium phosphate phases^1^,^2^. Prenucleation clusters are stable calcium triphosphate complexes generated from a supersaturated calcium and phosphate ion-containing solution that aggregate into branched structures and transform into amorphous calcium phosphate (ACP)^3^,^4^. Poly(anionic) acids resembling the polycarboxyl groups of non-collagenous proteins involved in biomineralization^5^,^6^,^7^ have been used as biomimetic analogs to stabilize ACP into a dense liquid phase^8^ consisting of polymer-induced liquid precursors^9^,^10^. These negatively-charged nanoparticles, attracted by regional net positive charges along the surface of collagen molecules, infiltrate the water compartments of collagen fibril. By using collagen fibrils as a mineralization template, the ACP condense within the gap zones and intrafibrillar spaces into apatite crystallites^11^,^12^,^13^.

Translation of contemporary biomineralization concepts into practical use is hampered by the difficulty to reproduce the condition in which *in vitro* mineralization is accomplished in solution with unlimited supply of biomineralization components. Instead of relying on the release/assembly of these components, it is necessary for translation systems to employ a strategy that delivers pre-formed poly(anionic) acid-stabilized ACP (Pa-ACP) as intermediate precursors for *in-situ* biomineralization. This may be achieved by using a porous delivery vehicle with large specific surface area and pore volumes for sustained release of intermediate precursors.

Mesoporous silica nanoparticles (MSNs) have been used experimentally as hosts for quantum confinement of guest molecules, as well as “tunable carriers” for drug delivery and controlled-release of gene-transfection agents^14^. Silanol groups on the surface of MSNs may be used for grafting of functional groups to establish a variety of molecular anchors^15^. The pore structure and large pore volume of MSNs are advantageous for accommodating large molecules such as proteins and RNAs^16^,^17^. This material may be used as delivery system for sustained release of mineralizing precursors, which can be used for *in-situ* remineralization of bone and teeth. Here, we report the use of amine-functionalized mesoporous silica nanoparticles (AF-MSNs) as delivery devices for fluidic Pa-ACPs. The physicochemical properties of AF-MSN and Pa-ACP-loaded AF-MSN and the release kinetics of Pa-ACP were characterized. The hypothesis tested was that AF-MSNs may be used as storage devices for release of intermediate precursors of collagen biomineralization. The use of Pa-ACP loaded AF-MSNs in collagen biomineralization was validated using a two-dimensional (2-D) reconstituted collagen mineralization model and a three-dimensional (3-D) rat tail tendon collagen model.

## Materials and Methods

All chemical reagents were purchased from Sigma-Aldrich (St. Louis, MO, USA).

### Mesoporous silica nanoparticles (MSNs)

Ammonium hydroxide solution (28%) was mixed with 1.81 mM cetyltrimethyl ammonium bromide solution (CTAB, Mw 364.45) at a volume ratio of 1:62.5. Tetraethyl orthosilicate (TEOS) was added drop-wise to the NH_4_OH-CTAB solution at a volume ratio of 1:338.67. After stirring for 4 hours at room temperature, the mixture was centrifuged to obtain a white precipitate. The precipitate was triple-washed with deionized water and ethanol, air-dried overnight and calcined at 500 °C for 3 hours to remove the CTAB template from the MSNs.

### Amine functionalized-MSNs (AF-MSNs)

Three hundred mg of template-free MSNs were dispersed in 60 mL of absolute ethanol. 3-aminopropyltriethoxysilane (APTES, Mw 221.37, 0.6 mL) was added drop-wise to the dispersed MSNs. After stirring at room temperature for 20 hours, the suspension was centrifuged to retrieve the white precipitate. The latter was triple-washed with ethanol under sonication and oven-dried at 65 °C overnight to obtain AF-MSNs.

### Poly(acrylic) acid stabilized amorphous calcium phosphate solution (Pa-ACP)

Equal volumes of 9 mM CaCl_2_·2H_2_O and 4.2 mM K_2_HPO_4_ aqueous solutions were prepared. Each solution was adjusted to pH 7.4 using Tris-buffered saline. Polyacrylic acid (Mw 1800; 200 μg/mL) was used as a biomimetic analog of extracellular matrix proteins involved in biomineralization, to stabilize the supersaturated calcium and phosphate ion-containing solution in the form of Pa-ACPs. The polyacrylic acid was added to the calcium ion-containing solution before mixing with an equal volume of the phosphate counter-ion solution.

### Pa-ACP loaded AF-MSNs

Two hundred milligram of AF-MSNs were suspended in 50 mL of Pa-ACP containing solution and stored at room temperature for 2 days to enable the polymer-induced liquid precursors to infiltrate the mesopores within the AF-MSNs. At the end of the infiltration period, the white precipitate was retrieved, rinsed three times with deionized water, filtered and stored as wet specimens at −20 °C until use.

### Collagen mineralization with Pa-ACP loaded AF-MSNs

#### Two-dimensional (2-D) collagen mineralization model

The ability of the AF-MSN delivery system to release Pa-ACPs to mineralize fibrillar collagen was firstly examined using a 2-D model of reconstituted collagen. This 2-D mineralization model permits expedited examination of the results of biomineralization by transmission electron microscopy (TEM). Collagen reconstitution was performed using 400-mesh carbon-and-formvar-coated Ni TEM grids (Electron Microscopy Sciences, Hatfield, PA, USA). The grids were placed over a 0.2 mg/mL collagen/acetic acid solution prepared from bovine skin-derived type I collagen lyophilized powder. Self-assembly of collagen fibrils was achieved by increasing the pH of the collagen solution with 1% ammonia vapor.

Fifty mg of Pa-ACP loaded AF-MSNs was suspended in 1.5 mL of 10 mM 4-(2-hydroxyethyl)-1-piperazineethanesulfonic acid (HEPES) buffer (pH 7.4) and placed in each well of a 24-well plate. A grid covered with reconstituted collagen was floated upside down on top of the mixture in each well. The assembly was incubated in a 100% humidity chamber at 37 °C for 2 days. Grids were rinsed with deionized water to remove excessive amount of AF-MSNs and air-dried for TEM examination at 100 kV.

#### Three-dimensional (3-D) collagen mineralization model

Rat tail tendon fascicles enveloped by their peritendinea were placed in iso-osmotic saline (0.9% NaCl) from the tails of mature (70 day-old) Wistar rats. The tendon fascicles were then incubated in the aforementioned HEPES buffer containing Pa-ACP loaded AF-MSNs at 37 °C for 7 days. The mineralized specimens were fixed in 2% glutaraldehyde, post-fixed in 1% osmium tetroxide, dehydrated in an ascending ethanol series (50–100%), immersed in propylene oxide and embedded in epoxy resin. Eighty nanometer thick sections were prepared and examined using the JSM-1230 TEM at 110 kV. All sections were examined without further staining. Selected area electron diffraction (SAED) was performed to identify the crystallinity of the infiltrated minerals.

### Analytical methods

The analytical methods used in this work include X-ray photoelectron spectroscopy (XPS), attenuated total reflection–Fourier transform infrared spectroscopy (ATR-FTIR), thermogravimetric analysis (TGA), solid-state nuclear magnetic resonance spectroscopy (NMR), powder X-ray diffraction (XRD), zeta potential measurement, TEM, scanning transmission electron microscopy-energy dispersive X-ray analysis (STEM-EDX), nitrogen adsorption-desorption analysis, atomic force microscopy (AFM), inductive coupled plasma-atomic emission spectroscopy (ICP-AES) and spectrophotometric determination of Ca, P and Si concentration. Full experimental details are given in the Supplementary Information S1.

## Results and Discussion

**Figure 1:**
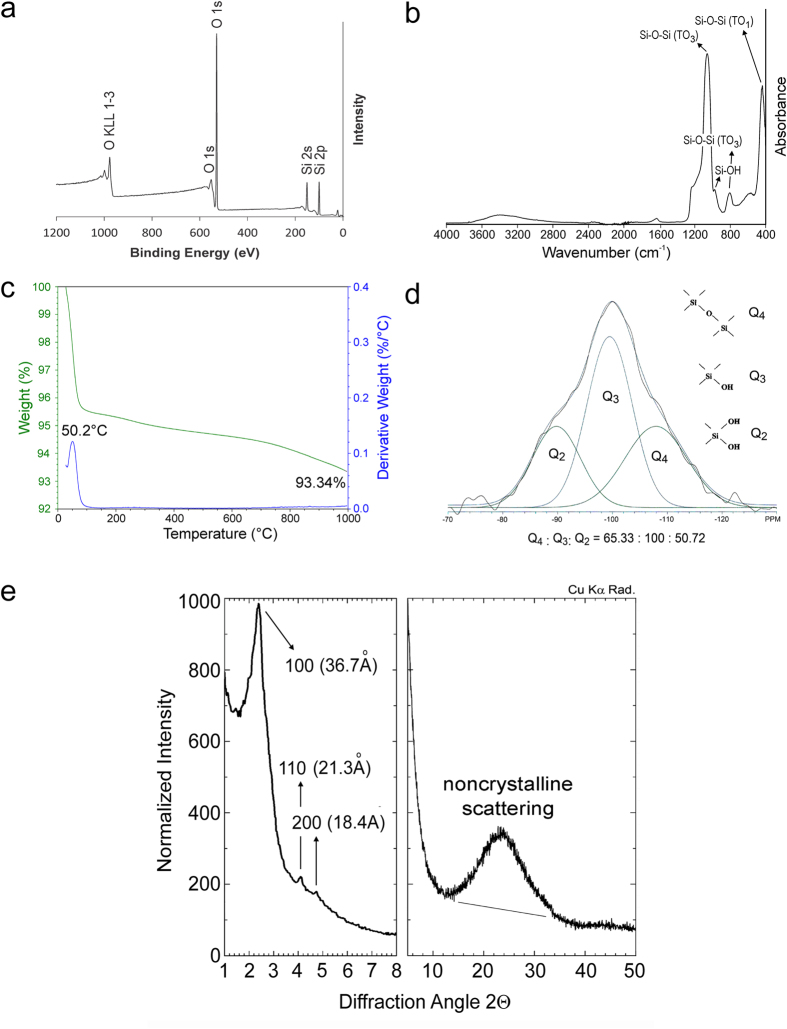
**a**) XPS survey scan of the elemental composition of template-free MSNs. **b**) Infrared spectrum of template-free MSNs. The Si-O-Si vibrational mode of silica was detected around 1070, 800 and 460 cm^−1^. The lowest frequency mode (460 cm^−1^) is assigned to transverse optical rocking motions (TO_1_ mode). Near 800 cm^−1^, a weak band due to Si-O-Si symmetric stretching (TO_2_ mode) can be observed. The highest frequency mode around 1070 cm^−1^ is assigned to the anti-symmetric stretching of the Si-O-Si bonds (TO_3_ mode). Si-OH vibrations near 940-960 cm^−1^ indicates the retention of silanol groups. **c)** TGA of template-free MSNs. The derivative weight loss peak at 50.2 °C represents the loss of physisorbed water. **d**) ^29^Si CP-MAS NMR spectrum of template-free MSNs. Deconvoluted peaks at −89.0, −99.0 and −107.9 ppm are assigned respectively to the Q_2_, Q_3_ and Q_4_ units (Q-series, Si(OSi)_x_(OH)_4-x_) originating from mesoporous silica. Q_4_: siloxane bridges; Q_3_: single silanols; Q_2_: germinal silanols^18^. **e**) Small-angle (left) and wide-angle (right) XRD of template-free MSNs. Diffraction peaks at (100), (110) and (200) are characteristic of a 2-D hexagonal lattics (*p6* mm)^19^. Non-crystalline scattering identified with wide-angle XRD is characteristic of the amorphous state of the mesoporous silica nanoparticles.

Because commercially available mesoporous silica particles are too large for the purpose of the present study (>250 nm diameter), MCM-41 (Mobil Composition of Matter No. 41) type MSNs with particle diameter below 100 nm were first synthesized by a sol-gel process, using TEOS as the silicon source, CTAB as surfactant and water as solvent. The nanoparticles were calcinated at 500 °C to remove the surfactant template. Chemo-analytical characterization of the template-free MSNs was performed using XPS, ATR-FT-IR, TGA, NMR and XRD (Fig. 1). The XPS survey spectrum showed that template-free MSNs were composed exclusively of silicon and oxygen. The infrared spectrum exhibited peaks that are characteristic of the Si-O-Si bond and silanol, with no evidence of remnant surfactant. This was also evident from TGA, with an overall weight loss of 6.6 wt% that was attributed to removal of physisorbed water. Profused silanol groups in the MSNs was evident from the deconvoluted ^1^H → ^29^Si cross polarization-magic angle spinning NMR spectrum, with a ratio of 65.33: 100: 50.72 for the relative distribution of siloxane bridge (Q_4_), single silanol (Q_3_) and germinal silanol (Q_2_)^18^. Wide and small angle XRD showed that MSNs were amorphous with a 2-D hexagonal mesopore structure^19^.

**Figure 2:**
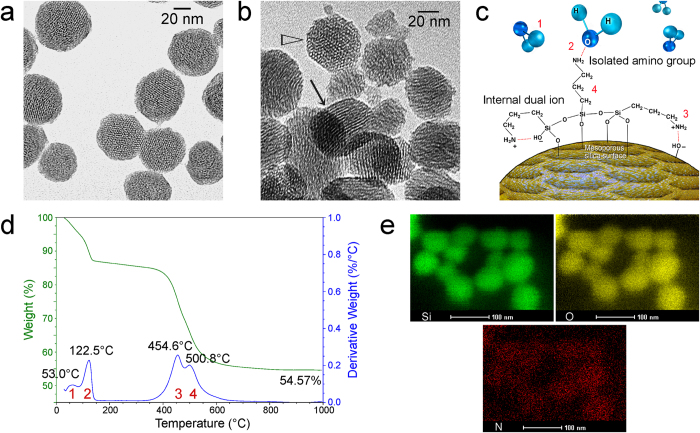
**a**) TEM of unstained MSNs prior to amine functionalization. **b**) TEM of unstained AF-MSNs. Arrow: mesoporous nanochannels seen in parallel arrangement. Open arrowhead: cross-sectional view of the mesoporous nanochannels. **c)** Schematic illustration of the assignments of the four TGA derivative weight-loss peaks. **d**) TGA data shows an overall weight loss of 45.4 wt%, with 4 discernible derivative weight-loss peaks. **e**) STEM–EDX mapping of AF-MSNs shows elemental distribution of Si, O and N within the nanoparticles.

Although these MSNs were capable of loading Pa-ACP via capillary imbibition^20^, this is not an efficient controlled-release system because both Pa-ACP (−32.91 ± 1.32 mV) and MSNs (−12.36 ± 0.08 mV) have negative zeta potentials, which render the release of Pa-ACP too fast in the absence of electrostatic attraction (Supporting Information S2). To enable effective uptake of Pa-ACPs and their efficient release, template-free MSNs were amine-functionalized by post-synthesis grafting with APTES^21^,^22^ to produce AF-MSNs with a positive zeta potential (18.41 ± 1.34 mV; Supporting Information S2). The TEM image of the parent MSNs (Fig. 2a) indicate that they are ultrastructurally similar to the AF-MSNs (Fig. 2b) in exhibiting uniform, parallel mesoporous channels. The results of TGA (Fig. 2d) shows an overall weight loss of 45.4 wt%, with 4 discernible derivative weight-loss peaks. A schematic illustration of the assignments of the four TGA derivative weight-loss peaks is shown in Fig. 2c. These four weight-loss peaks correspond to loss of physisorbed water (53.0 °C), dissociation of hydrogen-bonded (chemisorbed) water from isolated functionalized aminopropyl groups (122.5 °C), dissociation of internal hydrogen bonding from interaction of aminopropyl groups with MSN silanol groups, dehydroxylation of the silica surface or internal pore silanols and decomposition of organic functional groups (454.6 °C and 500.8 °C) (Fig. 2d)^23^. These processes may occur from both the silica surface and pore walls. The STEM–EDX mapping of AF-MSNs shows elemental distribution of Si, O and N within the nanoparticles (Fig. 2e). The presence of N signals confirms that amine groups were incorporated into AF-MSNs.

**Figure 3:**
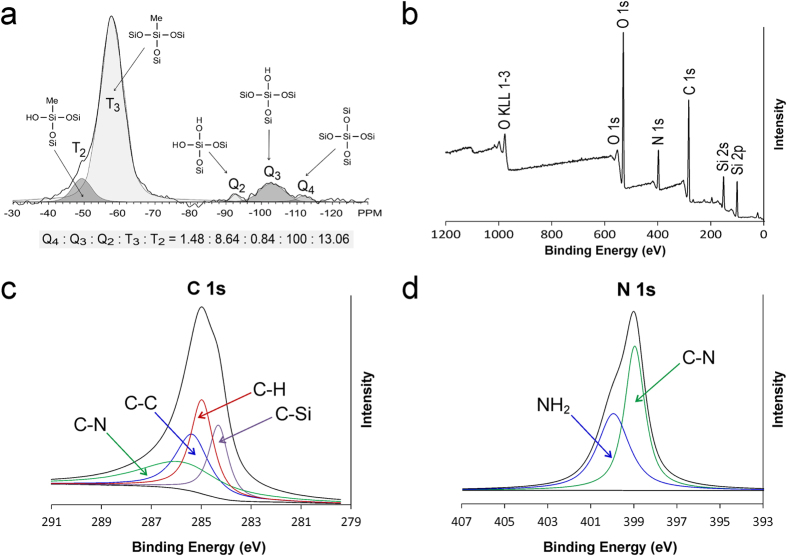
a) ^29^Si CP-MAS NMR spectrum of AF-MSNs. **b**) Wide-scan XPS spectrum showing the elemental composition of AF-MSNs and the chemical bonding states of aminopropyltriethoxysilane (APTES) and mesoporous silica. **c)** High resolution XPS spectrum of C1s. **d**) High resolution XPS spectrum of N1s.

Amine functionalization of MSNs was further verified using ATR-FTIR (Supporting Information S3)^24^, ^1^H → ^29^Si CP-MAS NMR^25^ (Fig. 3a) and XPS (Fig. 3b–d)^26^,^27^,^28^. The FT-IR results demonstrated presence of C-H_2_ (2883 and 2850 cm^−1^), N-H_2_ (1564 and 1481 cm^−1^) and N-H bands (3270 cm^−1^) in AF-MSN, which confirmed that APTES was grafted to the parent MSNs. ^29^Si CP-MAS NMR spectrum of AF-MSNs showed deconvoluted peaks at −89.0, −99.0 and −107.9 ppm. They are assigned respectively to the Q_2_, Q_3_ and Q_4_ units (Qx, Si(OSi)x(OH)_4−x_) originating from inorganic silica. Peaks around −59 ppm (T_2_) and −67 ppm (T_3_) represent Si atoms originating from trialkoxysilane. Wide-scan XPS spectrum showed the elemental composition of AF-MSNs and the chemical bonding states of APTES and mesoporous silica. The deconvoluted peaks at 284.61 eV, 285.30 eV and 286.38 eV from high resolution XPS spectrum of C1s are assigned to the C-H, C-C and C-N moieties, respectively. The peak at 284.31 eV may be assigned to C-Si due to incorporation of the -Si-(CH_2_)_3_-NH_2_ bond after APTES functionalization. The deconvoluted peak at 399.95 eV in the high resolution XPS spectrum of N1s is attributed to the free amine group (−NH_2_), while the peak at 398.96 eV may be ascribed to the C-N bond derived from APTES.

**Figure 4:**
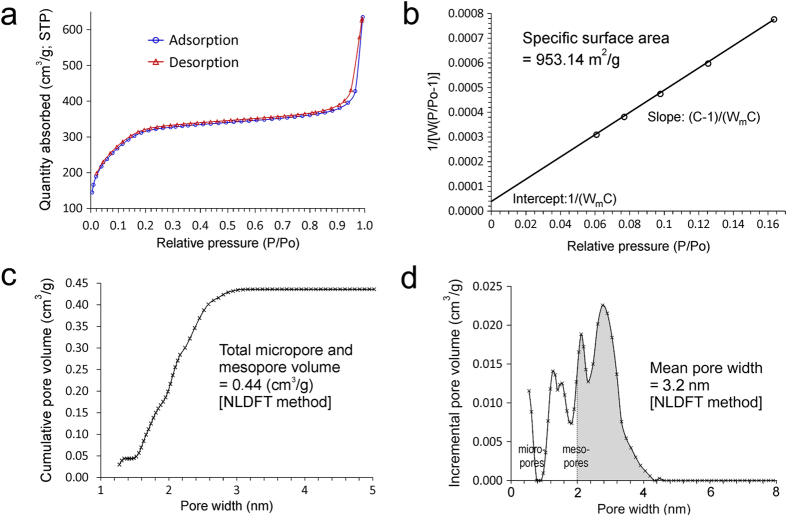
**a**) Nitrogen sorption of AF-MSNs showing type IV adsorption-desorption isotherms. **b**) Specific surface area of AF-MSNs. **c**) Total micropore and mesopore volume in AF-MSNs. **d**) Pore size distribution in AF-MSNs.

Nitrogen sorption of AF-MSNs (Fig. 4) yielded type IV adsorption-desorption isotherms characteristic of mesoporous silica; hysteresis in the multilayer range of the sorption isotherm was associated with capillary condensation within mesoporous structures^29^. The specific surface area of AF-MSNs, as determined using the Brunauer–Emmett–Teller method, was 953.14 m^2^/g. The non-local density functional theory was used as a model to investigate pore volume and pore size distribution, since it provides more accurate information on the micropores and mesopores when compared to methods based on the Kelvin equation^30^. The AF-MSNs have a pore volume of 0.44 cm^3^/g, consisting of both micropores (<2 nm) and mesopores (>2 nm), with a mean pore diameter of 3.2 nm. The large specific surface area and pore volume of AF-MSNs favor their use for loading of Pa-ACP.

**Figure 5:**
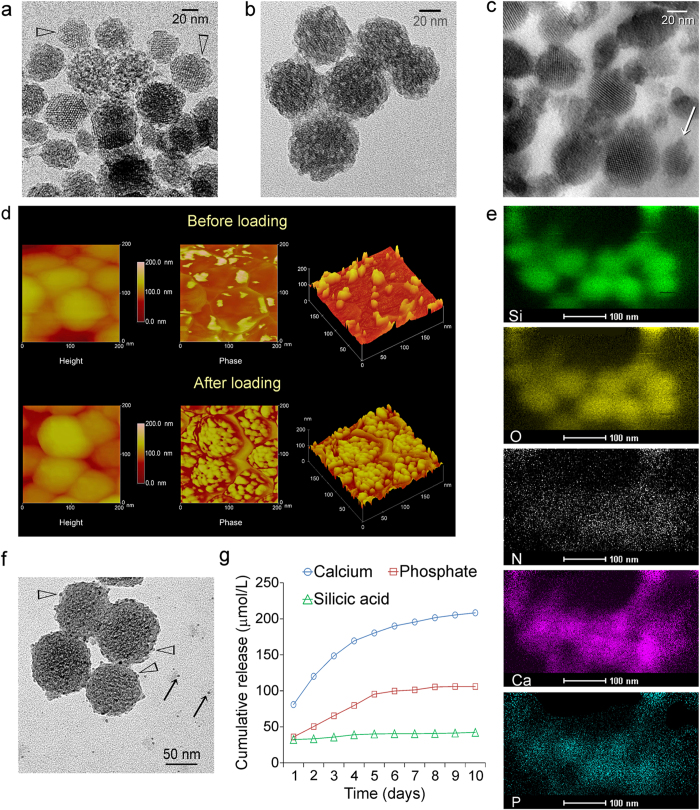
**a**) Unstained TEM image of unsectioned, template-free AF-MSNs with partially-loaded Pa-ACPs (arrowheads) revealing their mesoporous structure. **b**) Unstained TEM image of unsectioned AF-MSNs that were fully loaded with Pa-ACPs. **c**) Unstained TEM image of sectioned, epoxy resin-embedded Pa-ACP loaded AF-MSNs showing the presence of Pa-ACPs within the mesopores and around the periphery of the mesoporous silica nanoparticles (arrow). **d**) AFM height and phase images and three-dimensional presentation of the surface morphology AF-MSNs before and after loading of Pa-ACPs. Before loading, AF-MSNs have relative smooth surface profiles while the phase-contrast image and 3-D surface plot reveal localized regions that exhibit changes in viscoelastic properties or adhesion forces that may be contributed by anchoring of APTES on the MSN surface. After loading, the AF-MSNs have highly granular surface morphology. **e**) STEM-EDX mappings showing the different elements present within the loaded nanoparticles. **f**) Unstained TEM image showing release of Pa-ACPs (arrows) after immersion in HEPES buffer solution. Some of the Pa-ACPs were present on the surface of the AF-MSNs (arrowheads). **g**) Release kinetics of calcium, phosphate and silicic acid ions from Pa-ACP loaded AF-MSNs within a 10-day period after immersion in HEPES at pH 7.4.

The ability of template-free AF-MSNs to carry and release Pa-ACP is illustrated in Fig. 5. The Pa-ACP was prepared by using 200 μg/mL polyacrylic acid^7^ to stabilize ACP generated from a supersaturated solution of CaCl_2_·2H_2_O and K_2_HPO_4_. This stabilization process enabled ACP nanoparticles to be imaged in a desiccated state using conventional TEM without resorting to cryo-TEM^31^. This is analogous to the stabilization achieved in larger versions of zirconia-modified ACP; the zirconia prevents auto-transformation of the dried ACP powder into apatite^32^. Loading of Pa-ACP was performed by suspending AF-MSNs in a Pa-ACP containing solution for 2 days. After centrifuging, the Pa-ACP loaded AF-MSN precipitate was rinsed and stored at −20 °C. Because of their initial fluidic nature, it is possible for the Pa-ACP to initially infiltrate the internal pore channels of AF-MSNs (Fig. 5a). Additional Pa-ACP was attached to the surface of the AF-MSNs, completely masking their mesoporous ultrastructure (Fig. 5b). Tranmission electron microscopy of a 50 nm thick section of epoxy resin-embedded Pa-ACP loaded AF-MSNs shows the presence of Pa-ACP within the mesopores as well as on the surface of the sectioned silica nanoperticles (Fig. 5c). Two-dimensional AFM phase image and 3-D surface plots of unloaded AF-MSNs revealed relatively smooth surface morphology, whereas the surface of Pa-ACP loaded AF-MSNs was granular, with ACP clusters present on the nanoparticle surface (Fig. 5d). Elemental mapping using STEM-EDX indicated that Si, O, N, Ca and P were present in the Pa-ACP loaded AF-MSNs (Fig. 5e). When Pa-ACP loaded MSNs and AF-MSNs were subjected to microwave-assisted silicon digestion and analyzed using ICP-AES, the concentration of Ca, P and Si in Pa-ACP loaded AF-MSNs were 1.23, 0.58 and 32.28 wt% respectively. In comparison, the concentration of Ca, P and Si in Pa-ACP loaded MSNs was 0.06, <0.05 and 43.27 wt% respectively. Based on these information, it is speculated that physisorption of Pa-ACP may be attributed to a combination of capillary action of the mesopores^33^, as indicated by the presence of Ca and P in Pa-ACP loaded, non-amine-functionalized MSNs, as well as electrostatic attraction between the oppositely-charged Pa-ACPs and amine functionalities in the MSNs^34^. Because the amount of Ca and P present in the loaded MSNs were far less than the amount present in AF-MSNs, we speculate that physisorption via electrostatic attraction plays a more important role in the loading of the Pa-ACPs than capillary action via surface wetting of the Pa-ACP with the surface of the mesoporous silica.

To investigate how intermediate precursors were released from AF-MSNs, Pa-ACP loaded AF-MSNs were dispersed in HEPES buffer solution for 10 days. Mesporous silica nanoparticles retrieved from the buffer after 2 days were examined by TEM. The released Pa-ACPs were manifested as electron-dense nanoparticles on the surface and in the vicinity of the AF-MSNs (Fig. 5f). Release of Pa-ACPs may be caused by displacement of the physisorbed Pa-ACP via competitive adsorption of the HEPES zwitterion^35^,^36^,^37^. Although the use of HEPES is far removed from a clinical scenario, it serves as the proof-of-concept that Pa-ACPs may be displaced from the AF-MSNs. *In vivo*, there are many available zwitterions available for competitive adsorption in collagen, for instance, phospholipids, phosphoproteins; even free amino acids in body fluids can serve as zwitterions. The cumulative release profiles of calcium, phosphate and silicic acid, as determined using spectrophotometric determination methods specific for the corresponding ions, are shown in Fig. 5g. Both calcium and phosphate ions were rapidly released during the first 6 days and became stabilized thereafter. The amount of silicic acid from the AF-MSNs was less compared with the calcium and phosphate ions.

**Figure 6:**
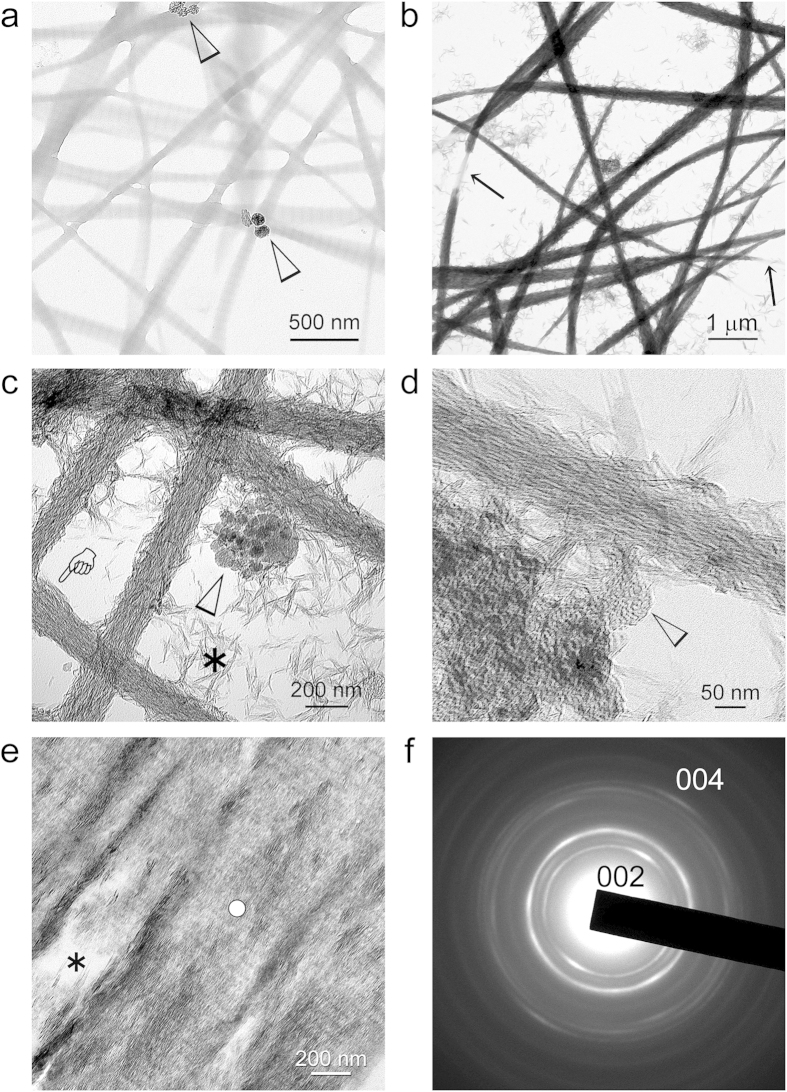
**a–d**) Unstained TEM images of collagen biomineralization in a 2-D model consisting of bovine skin collagen reconstituted on TEM grids.**a**) Grid placed over Pa-ACP loaded AF-MSN containing HEPES buffer for 15 min. AF-MSNs could be identified in the vicinity of the unmineralized collagen fibrils (arrowheads). **b**) Most of the fibrils were mineralized after 4 days. Arrows: Unmineralized portions of collagen fibrils **c**) High magnification of intrafibrillar (pointer) and extrafibrillar (asterisk) mineralization. Arrowhead: Pa-ACP loaded AF-MSN. **d**) Very high magnification showing arrangement of intrafibrillar mineral strands along the microfibrillar spaces, with no evidence of cross banding. Arrowhead: Pa-ACP loaded AF-MSN. **e**) Unstained TEM image of collagen biomineralization in a 3-D model consisting of natural collagen fibrils derived from rat tail tendon. Asterisk: unmineralized intrafibrillar regions after 7 days of mineralization. White circle: site from which electron diffraction was performed. **f**) Selected area electron diffractions of the crystalline deposition indicate that apatite crystallites are aligned along the longitudinal axis of the collagen fibril.

**Figure 7:**
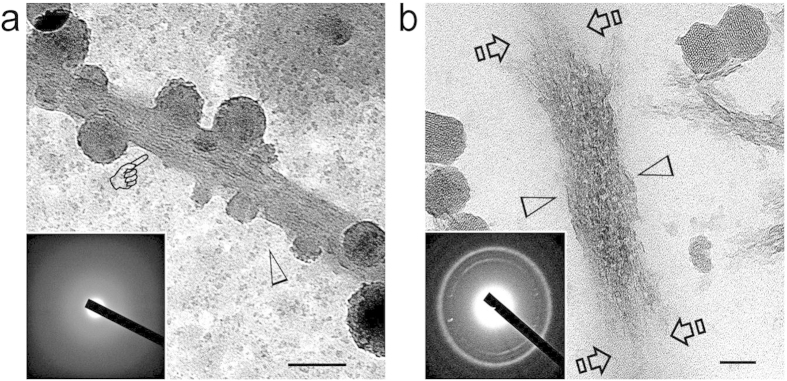
**a**) At 24 hours, unstained collagen fibrils were partially filled with electron-dense, amorphous minerals within their intrafibrillar spaces (pointer). Open arrowhead: calcium phosphate prenucleation clusters. Bar = 100 nm. Inset: SAED taken from the collagen fibril. **b**) After 2 days, collagen fibrils were partially mineralized by apatite crystallites (between open arrowheads). Regions within the same fibril that were not infiltrated by polyacid-stabilized amorphous calcium phosphate (Pa-ACP) are indicated by the open arrows. Mesoporosity of the AF-MSNs became apparent again after release of Pa-ACP. Bar = 50 nm. Inset: SAED taken from the partially-mineralized region of the collagen fibril.

The ability of Pa-ACP loaded AF-MSNs to mineralize collagen fibrils was first examined using a 2-D collagen mineralization model^6^,^7^. Bovine skin-derived type I collagen solution was allowed to self-assemble into fibrillar collagen on nickel TEM grids by interacting with ammonia vapor^31^,^38^. Mineralization of collagen fibrils was performed by floating TEM grids over a mineralization assembly consisting of Pa-ACP loaded AF-MSNs suspended in HEPES buffer at 37 °C for 1–4 days. Compared with the unmineralized control (Fig. 6a), most of the collagen fibrils were highly mineralized after immersion in the mineralization assembly for 4 days (Fig. 6b), although discrete unmineralized segments were observed within the highly mineralized collagen fibrils. Apart from intrafibrillar mineralization of the reconstituted collagen, extrafibrillar deposition of apatite clusters could also be identified, with a small amount of AF-MSNs attaching to the mineralized collagen fibrils (Fig. 6c). At high magnification, the mineralized collagen fibrils were devoid of cross-banding and consisted of continuous, intertwined rope-like mineral strands that replicated the microfibrillar arrangements of the collagen molecules (Fig. 6d). In specimens that were mineralized for shorter time periods, transition of the intrafibrillar minerals from an amorphous state to crystalline state could be observed (Fig. 7). In addition, the mesoporous architecture of the Pa-ACP loaded AF-MSNs re-appeared with progressively longer periods of mineralization, which is suggestive of unloading of the Pa-ACPs from the nanoparticles (Fig. 7b). It is speculated that once the Pa-ACPs are released from the AF-MSNs, they may remain in a liquid-like status similar to polymer-induced liquid precusors^5^,^9^, or in the form of calcium phosphate aggregates^6^ that enable them to infiltrate the intrafibrillar milieu of a collagen fibril by using the collagen fibril as a mineralization template^11^,^12^. Attraction of the Pa-ACPs may be achieved via electrostatic interaction between the negatively-charged -COOH groups of the biomimetic analog with net positive charges present on the surface of the collagen fibrils^11^,^39^. Nevertheless, it remains to be resolved whether the mechanism of infiltration of the Pa-ACPs into the collagen fibril is dependent upon charge interactions alone or if additional factors such as capillary forces are involved.

The hypothesis that AF-MSNs may be used as storage devices for release of intermediate precursors of collagen biomineralization was further tested by using a 3-D collagen mineralization model consisting of natural soft tissue collagen fibrils derived from the rat tail tendon. Fresh tendon fascicles were cross-linked as previously described, and immersed in the mineralization assembly for 7 days prior to preparation for TEM examination (Fig. 6e). Because the fibrils were densely oriented in a parallel manner, AF-MSNs were excluded from extrafibrillar spaces and there were also regions within a heavily mineralized fibril that were completely devoid of minerals. Unlike the reconstituted collagen fibrils in the 2-D model, cross-banding could be clearly identified even in heavily mineralized rat tail collagen, with the minerals appearing as discrete crystallites instead of continuous strands (Supporting Information 4). Selected area electron diffraction of the intrafibrillar minerals yielded arc-shaped diffraction pattern along the 002 plane is characteristic of ordered alignment of apatite crystallites along the C-axis of the fibrils (Fig. 6f). The observation that the mineralization process was not uniform in both the 2-D and 3-D models (i.e. all or none, rather than a progressive transition) is inexplicable: regions of heavy mineralization within a single fibril could be found adjacent to regions that were completely devoid of mineralization. Unlike collagen fibrils from the turkey leg tendon that mineralize with increasing age^40^, the collagen employed in the present 2-D and 3-D models are soft tissue collagen fibrils that do not mineralize under natural conditions. In the turkey leg tendon, the collagen crosslinks identified from the fully mineralized compartments were different from those present in the unmineralized compartments^41^. Although these natural collagen crosslinks are markedly different from the artificial amide linkages created by the zero-length carbodiimide crosslinking agent^42^, we speculate that the unmineralized regions within mineralized fibrils may be caused by insufficient crosslinking of the collagen molecules in those regions^38^ or insufficient mineralization period. This issue should be perused further to understand factors that control pathological mineralization of soft tissues in the human body.

## Conclusions

Mineralized collagen scaffolds designed for most tissue engineering are prepared by pre-mineralizing the scaffolds in solutions with an unlimited supply of biomineralization components, prior to their implantation into the body. By contrast, continuous replenishment of the mineralization medium is not possible for *in-situ* remineralization of hypomineralized body tissues, for example, after wound closure in the case of an osteoporotic surgical site, or after restoring the tooth with a dental filling in the case of hypomineralized dentin. The fact that the dense 3-D rat tail tendon collagen fibrils can be mineralized using Pa-ACP loaded AF-MSNs provides the proof-of-concept that intermediate precursors of calcium phosphate biomineralization may be pre-fabricated for loading and release. This represents an important advance in the translation of biomineralization concepts into regimes for *in-situ* remineralization of bone and teeth. Systematic examinations concerning the biocompatibility of the ACP precursor delivery system are necessary prior to their medicinal use.

## Additional Information

**How to cite this article**: Zhang, W. *et al.* Biomimetic Intrafibrillar Mineralization of Type I Collagen with Intermediate Precursors-loaded Mesoporous Carriers. *Sci. Rep.* **5**, 11199; doi: 10.1038/srep11199 (2015).

## Supplementary Material

Supplementary Information
