# Supplementary material for: Biomimetic Intrafibrillar Mineralization of Type I Collagen with Intermediate Precursors-loaded Mesoporous Carriers
Source: Sci Rep. 2015 Jun 8;5:11199. doi: 10.1038/srep11199 (PMC4459175; doi:10.1038/srep11199)
Supplement: Supplementary Information [file srep11199-s1.doc]

**Supplementary Information**

**Biomimetic Intrafibrillar Mineralization of Type I Collagen with Intermediate Precursors-loaded Mesoporous Carriers**

Wei Zhang^†^, Xiao-juan Luo^†^, Li-na Niu^†^, Hong-ye Yang, Cynthia K.Y. Yiu, Tian-da Wang, Li-qun Zhou, Jing Mao*, Cui Huang, David H. Pashley, Franklin R. Tay*

**Supporting Information**

**Table of Contents**

**S1** Materials and Methods

**S2** Zeta potential of polyacrylic acid-stabilized amorphous calcium phosphate (Pa-ACP), MSNs and amine functionalized-MSNs (AF-MSNs)

**S3**  Attenuated total reflection-Fourier transform infrared spectroscopy of 3-aminopropyltriethoxysilane (APTES), MSNs and AF-MSNs

**S4** High magnification unstained TEM image of biomineralization of rat tail collagen (3-D mineralization model)

**S5** Additional references

**S1 Materials and Methods**

*1. X-ray Photoelectron Spectroscopy (XPS)*

The surface composition of MSNs and AF-MSNs was determined using a Kratos Axis Ultra DLD spectrometer (Kratos Analytical Ltd., Manchester, UK) equipped with an aluminum Kα X-ray source (1486.6 eV). Photoelectrons were collected with a passing energy of 160 eV for the wide-ranged survey spectra and 20 eV for the scan spectra. The collection step was 1.0 eV for the wide-ranged survey scan, and 0.05 eV for the high resolution analyses at selected energy intervals. All analytical processes were conducted under ultra-high vacuum condition, which was maintained below 5×10^-9^ Torr. The binding-energy scale was calibrated according to the C 1s peak (285 eV) for adventitious carbon on the analyzed specimen surface. The Kratos charge neutralizer system was used for all specimens except for conductive specimens.

*2. Attenuated Total Reflection–Fourier Transform Infrared Spectroscopy (ATR-FTIR)*

A Nicolet 6700 FT-IR spectrophotometer (Thermo Scientific, Waltham, MA, USA) with an attenuated total reflection setup was used for analysis. Infrared spectra of aminopropyethoxy silane, MSNs and AF-MSNs were acquired between 4,000-400 cm^-1^ at 4 cm^-1^ resolution using 32 scans.

*3. Thermogravimetric Analysis (TGA)*

Thermogravimetric analysis was performed with a Q500 thermogravimetric analyzer (TA Instruments, New Castle, DE, USA). Approximately 25 mg each of template-free MSNs and AF-MSNs were placed in individual platinum pans and heated at a rate of 10 °C/min to 1000 °C in a nitrogen atmosphere. Data were analyzed using the Universal Analysis 2000 software (TA Instruments) and expressed as weight *vs* temperature as well as derivative weight *vs* temperature.

*4. Solid-State Nuclear Magnetic Resonance (NMR) Spectroscopy*

^29^Si solid-state NMR was employed at ambient temperature to examine the nuclear spinning properties of MSNs and AF-MSNs. A 270 MHz spectrometer (JEOL, Tokyo, Japan) was employed that was equipped with a 7 mm Magic Angle Spinning (MAS) probe. Spectra were acquired in the ^1^H→^29^Si cross polarization (CP) mode, using a MAS frequency of 4 kHz, with a 45 degree pulse angle of 5 μsec. The ^1^H Larmor frequency for ^29^S was 53.76 MHz. Chemical shifts were referenced to external tetramethylsilane at 0 ppm.

*5. Powder X-Ray Diffraction (XRD)*

X-ray diffraction of template-free MSNs was performed with a diffractometer (Rigaku D/max, Rigaku America, Woodlands, TX, USA). Copper Kα radiation (35 KeV, 65 mA) was used in the 2θ range of 1-90°. A step size of 0.025° and a 2θ counting step of 5 sec per step were employed. Determination of *d*-spacing values was based on Bragg-Brentano geometry.

*6. Zeta Potential*

The zeta potential of nanoparticle suspensions was measured with the electrophoretic light scattering method, using a DelsaNano C Zeta Potential Analyzer (Beckman Coulter, Inc., Brea, CA, USA). Analyses of Pa-ACP, MSNs and AF-MSNs suspended in Milli-Q water (pH adjusted to 7.4) were performed in duplicates using a flow cell at 25 °C.

*7. Transmission Electron Microscopy (TEM)*

The ultrastructure of AF-MSNs and Pa-ACP loaded AF-MSNs was examined using a JEM-1230 TEM (JEOL, Tokyo, Japan) at 110 kV. A small amount of each specimen was ultrasonically dispersed in ethanol (10 mg/mL), transferred to single-slot carbon-and-formvar-coated copper grids and examined unstained. Reconstituted collagen fibrils deposited on Ni grids was also examined unstained to avoid interference with the determination of their mineralization status. Crystallinity and crystal structure of the specimens were examined using selected area electron diffraction (SAED).

*8. Scanning Transmission Electron Microscopy-Energy Dispersive X-ray Analysis (STEM-EDX)*

Elemental compositions of AF-MSNs and Pa-ACP loaded AF-MSNs were investigated in specimens previously prepared for TEM using a Tecnai G2 STEM (FEI, Hillsboro, OR, USA) at 200 kV. Spectrum acquisition and elemental mapping were conducted using an Oxford Instruments INCA x-sight detector. Images were collected with a Gatan 1K x 1K CCD camera. Elemental mappings were acquired with the FEI TIA software using a spot dwell time of 300 msec. Drift correction was performed after the acquisition of every 30 images.

*9. N_2_ Adsorption-Desorption*

Nitrogen adsorption-desorption was used to characterize the specific surface area, pore volume and pore size distribution of AF-MSNs. Nitrogen sorption isotherms were measured at 77 K over the relative pressure (P/P_0_) range of 0.001-0.9, using a TriStar II 302 Surface Area and Porosity Analyzer (Micromeritics Corp., Norcross, GA, USA). Prior to analysis, an aliquot portion of specimen to be investigated was heat-conditioned for 3 hours at 200 °C under nitrogen flow. The Brunauer–Emmett–Teller (BET) method was used to calculate the specific surface areas. The non-local density functional theory (NLDFT) model was employed to estimate the total pore volumes of the micropores and mesopores within the specimens and their pore size distributions. This NLDFT model was a cylindrical model derived from the simulation of adsorption onto mesoporous silica and zeolite materials. Such a model has previously employed to analyze the pore size distribution in MCM-41 type MSNs following nitrogen adsorption.^[S1]^ Pore volume and pore size distribution were derived from the adsorption branch of the isotherms.

*10. Atomic Force Microscopy (AFM)*

The surface morphology of AF-MSNs before and after loading of Pa-ACPs were examined using a Digital Instruments Nanoscope IV (Veeco, NY, USA). Briefly, 0.01 g of each specimen was suspended in 15 mL of ethanol and dispersed ultrasonically for 1 hour. One drop of suspension was then placed on the surface of a clean, cleaved mica plate and dried at room temperature. Scanning was performed in the tapping mode, using a scan size of 200x200 nm and a scan rate of 1 Hz. Images were analyzed for height, phase contrast and surface plot using the Nanoscope 5.31r1 software (Veeco).

*11. Inductively Coupled Plasma-Atomic Emission Spectroscopy (ICP-AES)*

The total amount of Ca, P and Si present following loading of MSNs and AF-MSNs with Pa-ACPs was examined using ICP-AES. The silica-containing specimens were digested with 48-51% hydrofluoric acid using microwave-assisted digestion (Multiwave 3000 Microwave Digestion System, Anton Paar, Ashland, VA, USA). The digestion conditions were 1200 W maximum with a 5 min ramp, a 20-min holding time and a maximum infrared temperature of 170 °C. Room temperature specimens were brought up to volume by mass on a calibrated precision weighing balance and analyzed with a Perkin Elmer Optima 3000 DV ICP system (Perkin Elmer, Waltham, MA, USA).

*12. Spectrophotometric determination of Ca, P and Si concentration*

A UV-1800, UV-Vis Spectrophotometer (Shimadzu, Columbia, MD, USA) equipped with a pair of 1-cm quartz matched cells was used for all spectral measurements.

*12.1* Ca determination ^[S2]^

O-cresolphthalein complexon (OCPC) was selected as the chromogenic reagent and 8-hydroxyquinoline (8-HQ) as the masking agents for Mg. The OCPC solution was obtained by mixing 32.5 mg of OCPC, 1.1 g of 8-HQ, 250 mL of H_2_O and 7.5 mL of 37% HCl, and diluted with H_2_O to 500 mL. A calcium color developing agent was obtained by mixing OCPC solution, diethylamine solution (diethylamine:H_2_O = 21:500 v/v) and methanol as the ratio of 1.5:1.5:1. A calcium calibration curve was prepared by measuring the absorbance at 570 nm after adding 50 μL of CaCl_2_ (25, 50, 75, 100, 125 or 150 μg/mL) to 4 mL of the calcium color developing agent.

For determining the kinetics of calcium release, 100 mg of Pa-ACP loaded AF-MSNs was suspended in 3 mL of 4-(2-hydroxyethyl)-1-piperazineethanesulfonic acid (HEPES) buffer solution (10 mM, pH 7.4) at 37 °C. At different time periods (1, 2, 3, 5, 7 and 10 days), the mixture was centrifuged at 4000 rpm for 4 min. A 50 μL aliquot of the supernatant was used for determining the absorbance at 570 nm. Calcium concentration was determined from the pre-established regression equation.

*12.2* P determination ^[S3]^

The bismuth-phosphomolydate complex method was employed for determination of the phosphate release kinetics, based on the formation of the blue bismuth-phosphomolybdate complex. Fifty microliter of HClO_4_ solution (60%, t/t), 20 μL of ammonium heptamolybdate solution (0.12 mol/L), 40 μL of bismuth working solution (0.001 mol/L) and various volumes of a phosphorus standard solution (200 mg/L; prepared from KH_2_PO_4_) were placed in 1 mL centrifuge tubes. After adding 50 μL of ascorbic acid solution (0.2 g/L) and diluting with deionized water to 1 mL, the solution was allowed to stand for 10 min. A phosphorus calibration curve was prepared by plotting the absorbance obtained at 720 nm from different volumes of the phosphate standard solution. The initial straight line portion of the absorbance-concentration plot was used to establish a linear regression equation for subsequent determination phosphate release.

For determining the kinetics of phosphate release, 100 mg of Pa-ACP loaded AF-MSNs was suspended in 3 mL of HEPES buffer solution at 37 °C. At different time periods (1-10 days), the mixture was centrifuged at 4000 rpm for 4 min. A 200 μL aliquot of the supernatant was used for determining the absorbance at 720 nm. Phosphate concentration was determined from the pre-established regression equation.

*12.3* Si determination ^[S4]^

The silicomolybdic acid method was employed for spectrophotometric determination of Si in water, based on the ability of silicic acid to form yellow silicomolybdic acid in the presence of acidified ammonium heptamolydate. The reagents used for Si determination were: 26.86 mmol/L of tetrasodium ethylenediaminetetraacetate solution (Na_4_EDTA), 1.0 N HCl solution, 10 N NaOH solution, 1.35 mol/L sodium sulphite (Na_2_SO_3_) solution, 0.67 mol/L tartaric acid (H_2_C_4_H_4_O_6_) and 42.08 mmol/L ammonium molybdate ((NH_4_)_6_Mo_7_O_24_·4H_2_O) solution A Si calibration curve was prepared by adding 125 μL of 1.0 N HCl, 125 μL of diluted Na_4_EDTA solution and 125 mL of ammonium molybdate solution to 250 μL of silica working solutions prepared with different concentrations of silicic acid. The mixture was allowed to stand for 5 min and then 125 μL of tartaric acid solution was added. After allowing the mixture to stand for an additional 5 min, 250 μL of the prepared Na_2_SO_3_ solution was added. The final mixture was allowed to stand for at least 30 min and the absorbance of the colored solution was measured at 700 nm. The initial straight line portion of the absorbance-concentration plot was used to establish a linear regression equation for subsequent determination of Si release.

For determination of the kinetics of silicon release, 100 mg of Pa-ACP loaded AF-MSNs was suspended in 3 mL of HEPES buffer solution at 37 °C. At different time periods (1-10 days), the mixture was centrifuged at 4000 rpm for 4 min. A 200 μL aliquot of the supernatant was used for determining the absorbance at 700 nm. Silicon concentration was determined from the pre-established regression equation.



**S2 Zeta potential of Pa-ACPs, MSNs and AF-MSNs**


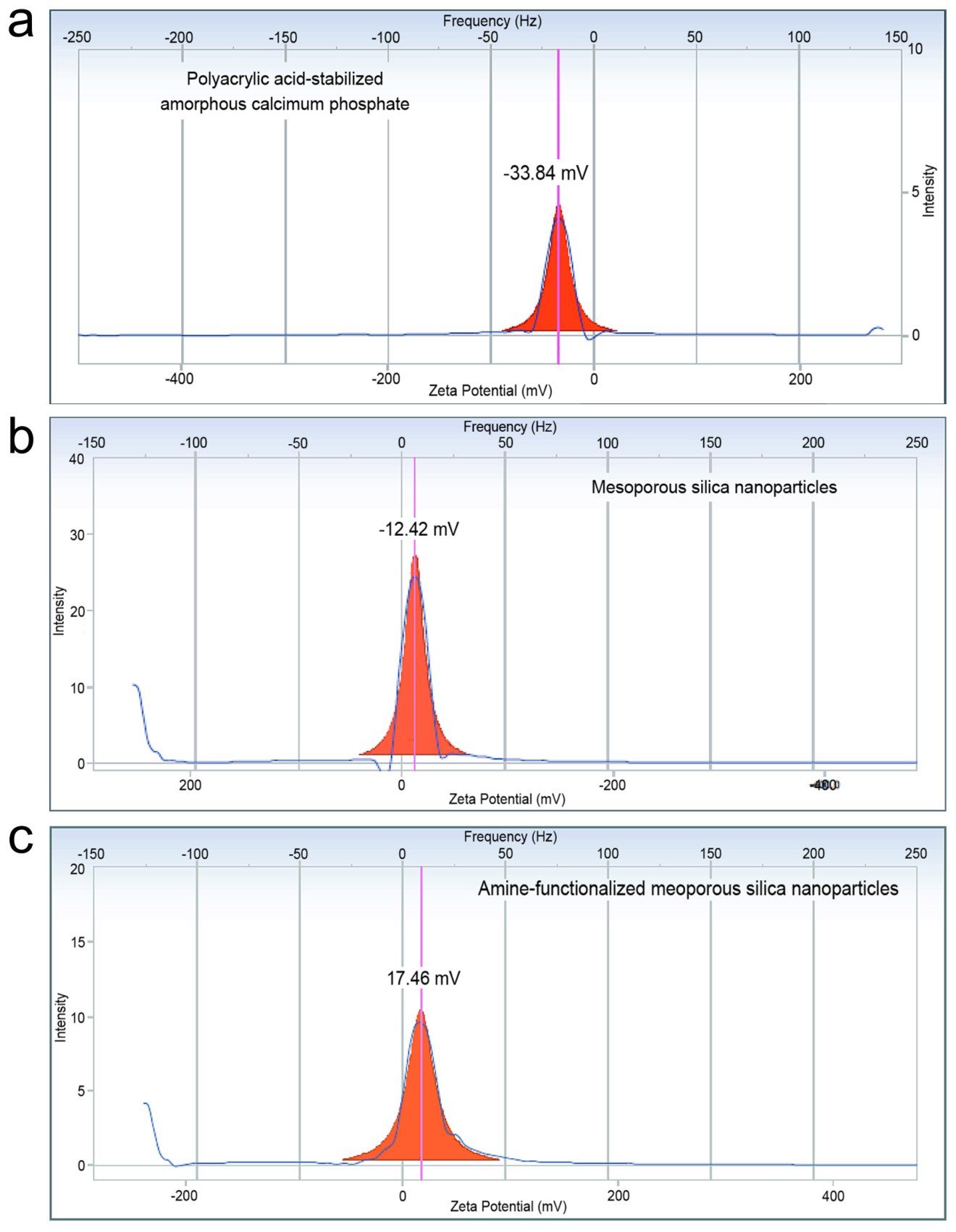


***Figure S2****.* Representative zeta potential measurements for **a)** Pa-ACP, **b)** MSNs ad **c)** AF-MSNs.

**S3 ATR-FTIR of APTES, MSNs and AF-MSNs**


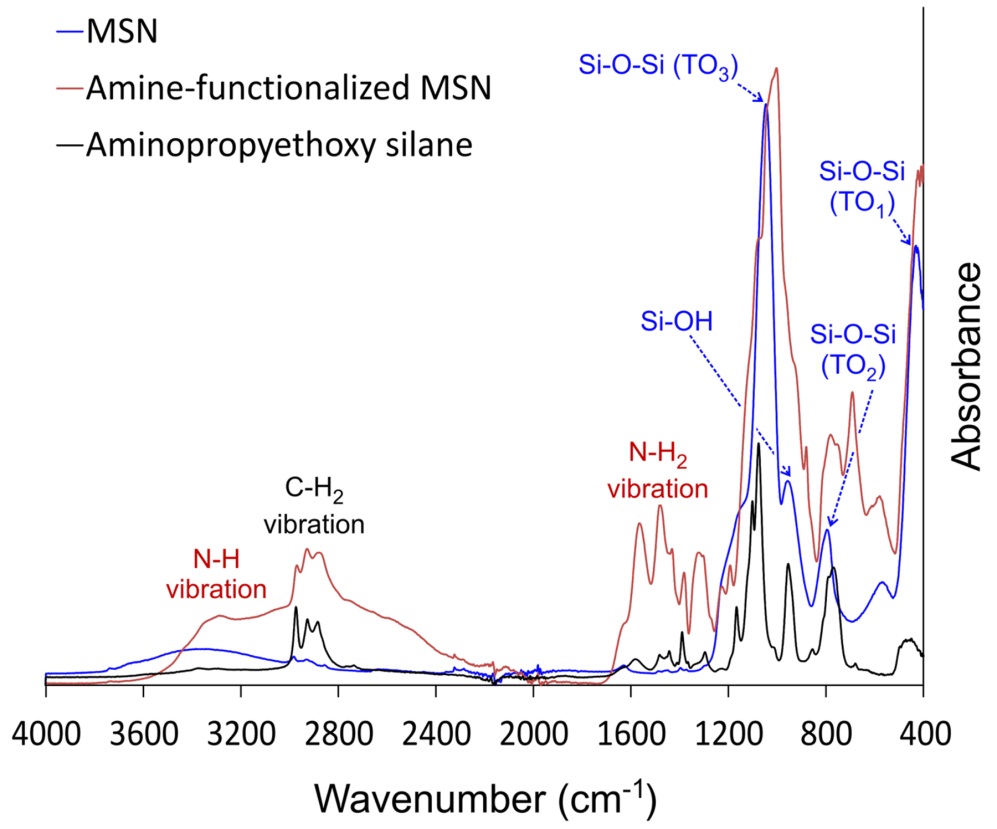


***Figure S3****.* Infrared spectra of MSNs, AF-MSNs and aminopropylethoxysilane (APTES). The Si-O-Si vibrational modes of silica detected around 1070, 800 and 460 cm^-1^ are absent from the spectrum of 3-aminopropyltriethoxysilane. The lowest frequency mode (460 cm^-1^) is assigned to transverse optical rocking motions (TO_1_ mode). Near 800 cm^-1^, a weak band due to Si-O-Si symmetric stretching (TO_2_ mode) can be observed. The highest frequency mode around 1070 cm^-1^ is assigned to the anti-symmetric stretching of the Si-O-Si bonds (TO_3_ mode).^[S5]^ The small band near 940-960 cm^-1^ is assigned to Si-OH vibrations.^[S6]^ The bands at 2883 and 2850 cm^-1^, assigned to C-H_2_ stretching from CH_2_CH_2_CH_2_-NH_2_ group derived from APTES, are absent from the parent MSNs. The bands which are indicative of N-H_2_ vibration (1564 and 1481 cm^-1^) and N-H vibration (at 3270 cm^-1^) cannot be observed in the parent MSNs.^[S7]^ Presence of C-H_2_, N-H_2_ and N-H bands in AF-MSN confirmed that APTES was grafted to the parent MSNs.

**S4 High magnification unstained TEM image of biomineralization of rat tail collagen (3-D mineralization model)**


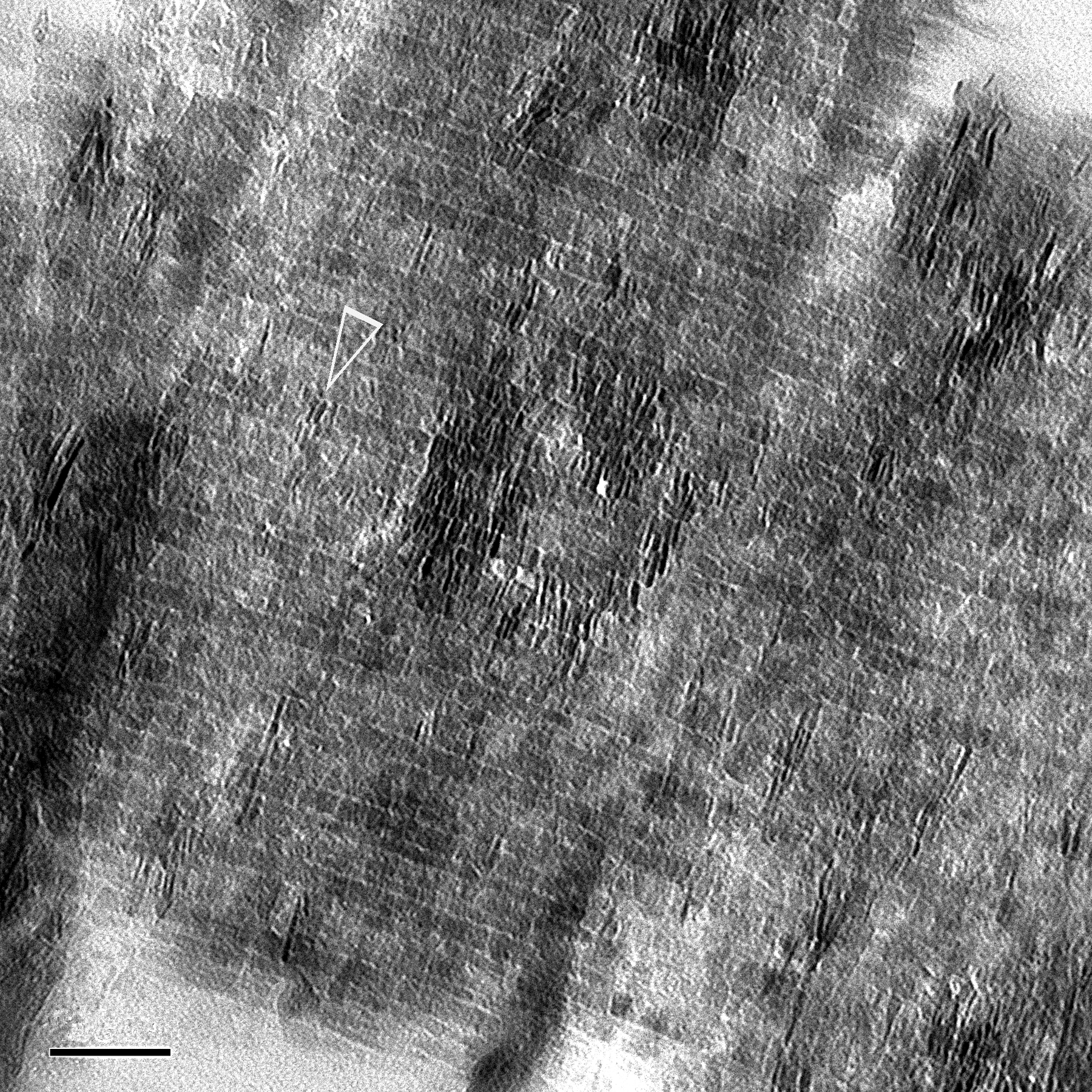


***Figure S4****.* Unlike the reconstituted collagen fibrils in the 2-D model, cross banding could be clearly identified even in heavily mineralized rat tail collagen, with the minerals appearing as discrete crystallites (open arrowhead) instead of continuous strands. Bar = 100 nm.

**S5 Additional references**

S1 Ravikovitch, P. I., Domhnaill, S. C. O., Neimark, A. V., Schueth, R. & Unger, K. K. Capillary Hysteresis in Nanopores: Theoretical and Experimental Studies of Nitrogen Adsorption on MCM-41. *Langmuir* **11**, 4765-4772 (1995).

S2 Nogueira, A. R. A., Brienza, S. M. B., Zagatto, E. A. G., Lima, J. L. F. C. & Araújo, A. N. Flow injection system with multisite detection for spectrophotometric determination of calcium and magnesium in soil extracts and natural waters. *J. Agric. Food Chem.* **44**, 165-169 (1996).

S3 Mihajlović, R. P., Kaljević, V. M., Vukašinović, M. P., Mihajlović, L. V. & Pantić, I. Đ. Spectrophotometric method for the determination of phosphorus in natural waters using the bismuthphosphomolybdate complex. *Water SA.* **33**, 513-518 (2007).

S4 Coradin, T., Eglin, D. & Livage, J. The silicomolybdic acid spectrophotometric method and its application to silicate/biopolymer interaction studies. *Spectroscopy* **18**, 567-576 (2004).

S5 Galeener, F. L. Band limits and the vibrational spectra of tetrahedral glasses. *Phys. Rev. B*. **19**, 4292 (1979).

S6 Almeida, R. M. & Pantano, C. G. Vibrational spectra and structure of silica gel films spun on c-Si substrates. *SPIE* **1328***,* 329 (1990).

S7 Su, F., Lu, C., Kuo, S. C. & Zeng, W. Adsorption of CO_2_ on Amine-Functionalized Y-Type Zeolites. *Energy Fuels* **24**, 1441-1448 (2010).
